# Cannabis, More Than the Euphoria: Its Therapeutic Use in Drug-Resistant Epilepsy

**DOI:** 10.7759/cureus.9299

**Published:** 2020-07-20

**Authors:** Keri-Ann R Buchanan-Peart, Geraldine I Oribhabor, Rhutuja V Khokale, Maxine L Nelson, Ivan Cancarevic

**Affiliations:** 1 Internal Medicine, California Institute of Behavioral Neurosciences & Psychology, Fairfield, USA; 2 Obstetrics and Gynecology, California Institute of Behavioral Neurosciences & Psychology, Fairfield, USA; 3 Neurology, California Institute of Behavioral Neurosciences & Psychology, Fairfield, USA

**Keywords:** cannabis, drug-resistant epilepsy, epilepsy, medical marijuana, adverse effects, therapeutic use, cannabidiol, pharmacology

## Abstract

A significant number of epilepsy patients are refractory to conventional antiepileptic drugs. These patients experience considerable neurocognitive impairments that impact their quality of life and ability to function independently. This need for alternative treatment has generated increased interest in cannabis use as a therapeutic option in these patients. This review seeks to analyze data presented on the pharmacology, safety, and efficacy of cannabis use in patients with drug-resistant epilepsy (DRE) and to propose any future recommendations regarding its use. PubMed was used to retrieve all published studies and articles which evaluated the use of cannabis in epilepsy. The two foremost phytocannabinoids of cannabis showing anticonvulsant properties are tetrahydrocannabinol (THC) and cannabidiol (CBD). Due to the psychoactive properties of THC, most studies focused on CBD use in these patients. The use of CBD as an adjunct resulted in decreased seizure frequency, and secondary benefits observed included improvement in mood, alertness and sleep. Adverse events (AEs) reported were drowsiness, diarrhea, increased transaminases and worsening of seizures. It can safely be concluded that there is a significant benefit in DRE patients using CBD as adjunctive therapy. However, further controlled and adequately powered studies are needed to assess the pharmacokinetics and impact of the long-term use of cannabis.

## Introduction and background

Approximately 70 million people worldwide are affected by epilepsy [1]. Its prevalence ranges from 0.5% to 1% in developed countries and even higher in developing countries [2]. Despite the vast availability of antiepileptic drugs (AEDs), 30% of these people have drug-resistant forms [1,3].

The International League Against Epilepsy (ILAE) describes drug-resistant epilepsy (DRE) as the failure of adequate trials of two tolerated and appropriately chosen and used AED schedules (whether as monotherapies or in combination) to achieve sustained seizure freedom [4]. Sustained seizure freedom occurs when the patient is seizure-free for more than one year or has sporadic seizures separated by a period three times the maximum interval between seizures before the treatment, whichever is longer [4]. About 7% to 20% of children have DRE, while 30% to 40% of adult patients remain refractory to pharmacological treatment [2]. This has a significant effect on cognitive and behavioral function and ultimately, the quality of life of these patients [5]. As a result, some of them seek alternate antiepileptic agents such as cannabis to achieve seizure freedom. Cannabis is a plant with different species; the most common being Cannabis sativa and Cannabis indica [6]. For many years, it has been used recreationally and increasingly in conditions such as multiple sclerosis, sleep disorders and epilepsy [6]. Cannabis is composed of over 100 molecules called phytocannabinoids, which have an affinity to mammalian cannabinoid receptors in the endocannabinoid system (ECS) [7]. This system regulates cortical excitability and its functioning is altered in epilepsy [7-9]. Two primary cannabinoid receptors include CB1 and CB2 [10]. CB1 receptors are expressed mostly in the central nervous system, while CB2 receptors are expressed mainly in the immune system and mesenchymal-derived central nervous system microglia [7]. The two primary phytocannabinoids are Δ9-tetrahydrocannabinol (Δ9-THC) and cannabidiol (CBD). THC is the psychoactive constituent, and CBD is non-psychoactive [7,10]. THC's psychoactive properties have limited its use clinically due to adverse side effects, so there has been more research exploring the therapeutic use of CBD.

CBD has been shown to have neuroprotective, anticonvulsant, anxiolytic, antipsychotic, and anti-inflammatory properties [11,12]. The exact anticonvulsant mechanisms of CBD are unknown [13]. It is thought to act indirectly on cannabinoid receptors and its actions are mediated through agonist and antagonist effects on ion channels, neurotransmitter transporters, and multiple 7-transmembrane receptors [13-15]. The anticonvulsant properties of CBD have been demonstrated in a few studies; however, most are either retrospective or small-scale observational studies [8]. A few randomized controlled trials (RCTs) were conducted, particularly in treatment-resistant children and adolescents with rare epilepsy syndromes such as Dravet (DS) and Lennox-Gastaut (LGS) syndromes [8,16]. Reduction in seizure frequency was demonstrated in these studies when CBD was used as adjuvant therapy to existing AEDs, which led to the U.S. Food and Drug Administration (FDA) recent approval of a highly purified CBD extract for use as adjuvant therapy in these patients [16,17]. Further studies have also revealed adverse events (AEs) associated with the use of CBD, which includes sedation, decreased appetite, increases in transaminases, and diarrhea, behavioral changes, skin rashes, fatigue, and sleep disturbances [18]. Its sedative properties were amplified with the associated use of clobazam, as it is believed that CBD causes inhibition of CYP2C19 [14,19]. Elevation of aspartate aminotransferase (AST) and alanine aminotransferase (ALT) levels was also seen, most notably in patients with associated valproate use [14,20-21].

While there is some research showing the efficacy of cannabis in DRE, there are factors limiting further study and implementation of its use. The legalization of cannabis use has been limited for many years, with only recent laws approving it for medical use. In the United States, half of the states have legalized cannabis for medical use. At the same time, 17 allow the use of products that are high in CBD and low in THC [22]. Also, there has been a lack of appropriately powered and high-quality studies evaluating its use [23-25]. In addition, the correct dosage and preparation of CBD were either unknown or not controlled in the majority of these studies [14]. Our full understanding of the pharmacokinetics of medical cannabis is thus deficient and curtails its use on a broader scale [8,10].

Hence this literature review seeks to evaluate the pharmacology, efficacy and adverse effects of the therapeutic use of cannabis in persons with DRE. It also aims to highlight any areas needing further clinical development to allow for more widespread use of cannabis in persons who have failed conventional antiepileptic therapy.

## Review

Pharmacology

The ECS, which modulates neuronal excitability, has been shown to be dysfunctional in patients with epilepsy [26]. This system comprises two receptors CB1 and CB2 and is activated by endogenous cannabinoids such as anandamide and 2-arachidonoyl glycerol (2-AG) when there is an excessive neuronal activity [27,28]. These endogenous cannabinoids act primarily on CB1 receptors and are believed to reduce glutamate transmission [29]. Disruptions in this system lead to a sustained seizure response, as demonstrated in a study by Wallace et al. [29]. The effects of endogenous cannabinoids on CB1 receptors were observed using rat pilocarpine models of acquired refractory epilepsy [29]. When these receptors were antagonized using the CB1-selective cannabinoid receptor antagonist, SR141716, a sustained seizure response was observed in the models as well as an increase in 2-AG levels [29]. Another study by Marsicano et al. demonstrated the modulation of neuronal excitability by the ECS by using mutant mice lacking CB1 expression in principal forebrain neurons [30]. Administration of the excitotoxin, kainic acid, stimulated an increase in anandamide levels in CB1 positive mice resulting in a reduction of neuronal excitation [30]. However, its administration in CB1 deficient mice resulted in excessive seizures [30].

These studies emphasize the role of the endocannabinoids in epilepsy and suggest a benefit to using therapeutic agents targeting this system. Cannabis, which is composed of phytocannabinoids (primarily THC and CBD), has thus been a drug of interest as its effect on the ECS may explain its anticonvulsant properties. Gaston et al. reported that THC, which is the psychoactive component of cannabis, has a partial agonist effect on CB1 and CB2 receptors [31]. A further study by Wallace et al. showed that CB1 receptors mediate the anticonvulsant effect of THC, while CBD was not shown to have a similar effect [32]. Cheung et al. found that the therapeutic use of THC as an anticonvulsant has been limited due to its psychoactive properties and the possibility of drug tolerance [26]. Its long-term safety is hence a cause for concern [26]. CBD, the non-psychoactive component, has a low affinity for CB1 and CB2 receptors [26]. Its anticonvulsant properties occur through a variety of mechanisms. A 2018 review by Gaston et al. suggested that CBD indirectly affects CB1 receptors by preventing the degradation of anandamide, thereby increasing its levels [14]. Iannotti et al. found that CBD activated and rapidly desensitized the transient receptor potential of vanilloid type-1 (TRPV1) in a dose-dependent manner [33]. This is clinically significant in patients with epilepsy, as Iannotti et al. suggested that there is evidence that TRPV1 may contribute to the onset and progression of some forms of epilepsy [33]. Other studies suggested possible CBD antiepileptic mechanisms, which include antagonist action at GPR55, targeting of abnormal sodium channels, blocking of T-type calcium channels, regulation of adenosine receptors, regulation of voltage-dependent anion-selective channel protein (VDAC1), and regulation of the release of tumor necrosis factor alpha [14,26].

These studies presented reliable evidence of the effect of cannabis on the ECS and its possible role as an anticonvulsant. A wide range of plausible mechanisms has been proposed with a particular focus on CBD. Further studies of these mechanisms could lead to a greater understanding of its efficacy in different clinical conditions resulting in widespread cannabis use.

Efficacy

While the anticonvulsant properties of cannabis have been known for several years, there was a lack of adequate clinical data supporting its therapeutic use. DRE prevalence consists of 30% of people who have epilepsy, which has led to increased interest and research on the efficacy and safety of cannabis use as an adjunct in these patients. In 2014, Maa and Figi reported a case of a young girl with DS [34]. After using a titrated dose of a high concentration CBD: THC strain of cannabis (called Charlotte's Web) coupled with her antiepileptic medication, it was reported that by her third month, she had a > 90% reduction in generalized tonic-clonic (GTC) seizures [34]. After 20 months of using this drug, she reported having only 2-3 nocturnal GTC seizures per month, having initially had in excess of 300 seizures per month prior to its use [34]. Further retrospective studies, which included small groups of patients, were also documented. One such study by Press et al. reviewed charts of 75 children and adolescents with epilepsy who were given oral cannabis extracts [35]. Of these patients, 57% reported improved seizure control, with 33% reporting > 50% seizure reduction [35]. Another notable finding was that the response to treatment was more favorable in patients with epilepsy syndromes (Dravet, Doose and Lennox-Gastaut) who had reduction rates of 23%, 0%, and 88.9%, respectively [35]. This finding mirrored another retrospective multicenter study by Tzadok et al. [36]. This study included a cohort of 74 patients with epilepsy resistant to >7 AEDs, and their response to cannabis oil treatment over an average of six months was observed [36]. Eighty-nine per cent (89%) of patients reported seizure reduction ranging from less than 25% to 100% [36]. More than 25% of seizure reduction was found in 66% of patients with epileptic encephalopathies, while only 45% of other study participants had a comparable result [36]. While these studies and others showed some therapeutic effect of cannabis in epilepsy, they lacked control groups, consistent dose administration and were inadequately powered. There was thus an ever-growing need for double-blind, RCTs. An early double-blind RCT by Cunha et al. in 1980, studied 15 adult patients with treatment-resistant temporal lobe epilepsy, who were treated with 200-300 mg of CBD or placebo daily over 18 weeks [37]. Of the CBD patients, 50% were almost seizure-free, while one patient showed no improvement [37]. On the other hand, 88% of the placebo group achieved no clinical improvement [37]. Since that time, there have been a few RCT conducted with most being conducted in children and young adults with rare epilepsy syndromes. This limitation was, in part, attributed to legal restrictions of cannabis, which is a Schedule 1 drug under the Controlled Substances Act due to its high potential for abuse and lack of medically accepted use in the United States at that time [22]. Nonetheless, there has been data presented from these few RCT studies which suggest a benefit to using cannabis as an adjunct to therapy. In 2018, Lattanzi et al. conducted a systematic review and meta-analysis of four trials involving 550 patients with LGS and DS refractory to AED [15]. Patients assigned to the treatment group were given either a 10 mg or 20 mg CBD dose [15]. The outcomes studied were changes in percentage and proportion of patients who experienced at least a 50% reduction in monthly seizure frequency [15]. Favorable results for CBD use showed that percentage point changes in seizure frequency between the CBD 10 mg group and placebo group and the CBD 20 mg group and placebo group were 19.5 (95% CI 8.1-31.0; p = 0.001) and 19.9 (95% CI 11.8-28.; p < 0.001), respectively [15]. A total of 37.2% of patients who received 20 mg of CBD and 21.2% in those who received the placebo (CI 1.07-2.88; p = 0.025) had at least a 50% reduction in seizure frequency as shown in Table 1 [15]. It was further concluded that a greater reduction in seizure frequency in patients with uncontrolled LGS or DS is associated with adjunctive CBD use [15]. A review article by Chen et al. of the GWPCARE trial series, reported that patients with LS and DS who were two years of age and older using adjuvant CBD had a 17%-23% reduction of seizure frequency when compared with placebo [38]. In 2018, the first cannabis-derived medication called Epidiolex, a highly purified CBD extract was given approval from the FDA for the treatment of seizures associated with LGS or DS in patients 2 years of age and older [38].

 

**Table 1:** Effect of CBD use in patients with drug-resistant epilepsy CBD, cannabidiol

Change in seizure frequency pooled average difference (percentage points)				> 50% Reduction in all types seizure frequency			Drug Withdrawal		
										CBD 10 mg vs Placebo	Confidence Interval	CBD 20 mg vs Placebo	Confidence Interval	CBD 20 mg	Placebo	Confidence Interval	CBD	Placebo	Confidence Interval
19.5	8.1-31.0 (p value = 0.001)	19	11.8-28.1 (p value <0.001)	37.20%	21.20%	1.07-2.88 (p value = 0.025)	11.10%	2.60%	1.55-8.12 (p value = 0.003)

The benefit of cannabis use in patients with DRE has been clearly demonstrated in these studies, the outcome of which was the achievement of more than 50% seizure reduction. This use was primarily noted with highly purified CBD, which led to FDA approval of its use as an adjunct and its reclassification by the Drug Enforcement Agent as a schedule V agent [19]. Another crucial finding was that there was a more significant benefit in patients with rare epilepsy syndromes such as LGS and DS compared to other syndromes. While we may conclude that CBD use was efficacious in these patients, the paucity of randomized, double-blind controlled studies cannot be ignored. The largest meta-analysis reviewed consisted of only four trials with a summed total of 550 patients. This limitation in sample size was reflected in the wide confidence intervals obtained despite statistically significant p values. Consequently, there is an apparent need for more RCTs with larger sample sizes to get more precise estimates of the effects of cannabis use. Most studies were also limited to younger participants with rare epileptic syndromes. Further studies, including other epileptic patient groups, are necessary for the general applicability of cannabis use in patients with DRE.

Safety and tolerability

In addition to analyzing the efficacy of any novel therapeutic agent, the AEs need to be monitored to weigh the risks and benefits of its use clinically. While studies show a proven benefit to the use of CBD as an adjunct, there have also been AEs reported. In the meta-analysis conducted by Lattanzi et al., CBD associated A.E.s included drowsiness, decreased appetite, diarrhea, and increased serum aminotransferases [15]. A total of 87.9% and 72.2% of patients treated with CBD and placebo experienced AEs, resulting in discontinuation of treatment in 8.9% and 1.8% in the treatment and control groups, respectively [15]. A retrospective review by Tzadok et al. of 74 children with DRE treated with CBD, reported that 7% of patients withdrew from the study due to AEs, which included aggravation of seizures [36]. Some of these AEs, such as drowsiness and elevated transaminases, were attributed to drug-drug interactions. In a review article by Gaston and Szaflarski, it was noted that the sedative properties of CBD were magnified in patients who were also taking clobazam, which is usually prescribed for patients with LGS [14]. This is thought to be due to CBD inhibition of CYP2C19, resulting in increased levels of the active metabolite of clobazam called N-desmethylclobazam [14]. It further noted that increased transaminase levels occurred in patients who were also taking valproate [14]. However, these elevations were resolved after continued drug use, as was observed by Devinsky et al., who suggested that the changes may have been due to short-lived metabolic stress on the liver [39,40].

AEs occurred in a higher number of patients taking CBD in comparison to the placebo groups. These events varied, ranging from mild to moderate to more severe manifestations such as seizure aggravation. Study withdrawal was commonly due to patients experiencing these events, which may lead to challenges with compliance in future patients. Despite the suggestion that drug-drug interactions may explain some of these events, there are still many other factors to consider. These include patient factors (e.g., age, race), dosage and dose frequency, long term usage, and CBD composition. Further research into these areas will provide excellent insight and knowledge and will better guide clinicians and patients alike when considering CBD as a therapeutic option.

Quality of life

The quality of life experienced by epilepsy patients and even more so those with DRE is significantly affected, commonly impacting their physical, mental, and social needs [41]. Several studies have reported beneficial effects from CBD use, such as improvement in alertness, mood, sleep, and language skills. In an observational study by Pesantez-Rios et al., 15 patients with DRE who used CBD were monitored for up to a year [42]. Caregivers reported that there were significant neurocognitive improvements in behavior (73%), language (60%), Sleep (50%), Appetite (43%), and Mood (100%) [42]. In 2017, Rosenberg et al. sought to measure the quality of life in a group of 48 DRE children taking CBD as reported by caregivers over 12 weeks [41]. The quality of life survey used was: The Quality of Life in Childhood Epilepsy (QOLCE). This survey focused on the following major domains: physical function, cognitive function, emotional well-being, social function, and behavior [41]. This study found an 8.2 +/- 9.9 point improvement in the quality of life of the study participants in all the observed domains [41]. Interestingly, the QOLCE score improvements did not correspond with AEs or changes in seizure frequency with CBD use [41]. Therefore, it was suggested that CBD's impact on the quality of life of these patients might be independent of the effect it has on seizure reduction [41].

The observed beneficial effects on the quality of life with CBD use in DRE patients make a compelling case for therapeutic use. This may help them lead more fulfilling lives and increase their ability to contribute to society rather than being considered a burden. These reports of quality of life improvement, however, were primarily based on caregiver feedback and not the patients themselves, limiting our interpretation of these results. Most studies also gave a cursory report of these benefits, reiterating the need for more double-blind RCT focusing on the quality of life in DRE patients taking CBD.

## Conclusions

The anticonvulsant properties of cannabis have been reported for several years; however, its use as adjunctive therapy in DRE has increased in recent years. Cannabis mediates the ECS, which affects neuronal excitability. This makes it a superior choice for the adjunctive treatment of DRE patients. Studies have shown that cannabis use, primarily its CBD component, resulted in at least 50% seizure reduction in these patients. Additional positive effects included improvements in mood, alertness, sleep, and language skills, which led to improved quality of life. AEs associated with cannabis use include drowsiness, decreased appetite, increased transaminases, and seizure aggravation. Despite these advancements, more clarity is needed in terms of pharmacokinetics, long-term use efficacy, and factors related to AEs to explore the therapeutic potential of cannabis use fully.
